# Reducing prescribing for patients with a primary diagnosis of personality disorder: Insights from a staff survey within an ongoing quality improvement approach

**DOI:** 10.1192/j.eurpsy.2026.11474

**Published:** 2026-07-31

**Authors:** H. Bath, A. Adesanya, S. Cutler, R. Mann, K. Kerrigan, P. Peddu, A. Vaida, A. Atta, P. Ved, H. Mahmood, C. Handy, A. Owen

**Affiliations:** 1 CWPT, Coventry; 2 CWPT, Warwickshire, United Kingdom

## Abstract

**Introduction:**

Excessive prescribing for individuals with personality disorder remains a significant challenge across mental health services. Although medication may offer short-term relief during crisis, long-term psychotropic use lacks evidence of effectiveness and may contribute to polypharmacy, adverse side effects, and reduced focus on psychological interventions. This quality improvement (QI) project aims to promote evidence-based, psychologically informed care and to reduce unnecessary prescribing for people with a primary diagnosis of personality disorder.

**Objectives:**

To understand staff views on prescribing practices for patients with a primary diagnosis of personality disorder, to inform ongoing QI interventions.

**Methods:**

Baseline prescribing data were collected from electronic health records, focusing on psychotropic medication use among patients with personality disorder. Subsequently, a cross-sectional anonymous online survey was distributed to all clinical staff between 31^st^ March and 9^th^ May 2025. Quantitative data were analysed descriptively, and qualitative comments were thematically reviewed.

**Results:**

A total of n=102 staff members across inpatient and community settings completed the survey, including nurses, doctors, psychologists, pharmacists, social workers, occupational therapists, psychotherapists, managers, and support staff. Two-thirds had over five years of experience working with this population. Most respondents (88%) were aware of clinical guidelines for managing personality disorder. Fifty-one percent rated their knowledge of medication-based treatments as good or excellent, compared with 76% for psychological treatments. Only 35% felt fully supported to provide appropriate care. Notably, 97% of respondents agreed that there are problems with the amount of medication prescribed, citing issues such as polypharmacy, off-label use, lack of non-pharmacological alternatives, and side effect burden. Perceived causes included over-reliance on medication, limited access to psychological therapies, resource constraints, and pressures related to managing risk. Staff identified potential solutions such as improved access to psychological interventions, enhanced training, clearer prescribing guidance, and stronger multidisciplinary collaboration.

**Conclusions:**

Staff across settings recognise challenges with current prescribing practices for individuals with personality disorder and express a strong desire for change. Findings highlight the need for system-wide support to embed psychologically informed care, expand access to non-pharmacological treatments, and strengthen multidisciplinary approaches. These insights will inform the next phase of this ongoing QI project, focusing on education, guideline implementation, and structured medication reviews.

**Disclosure of Interest:**

None Declared

